# The role of affective temperaments in self-care and medication adherence among individuals with bipolar disorder: a moderation analysis

**DOI:** 10.3389/fpsyt.2024.1443278

**Published:** 2024-09-11

**Authors:** Giulia Visalli, Grazia Longobardi, Anna Maria Iazzolino, Martina D’Angelo, Valeria Di Stefano, Pasquale Paribello, Luca Steardo, Mirko Manchia, Luca Steardo

**Affiliations:** ^1^ Psychiatry Unit, Department of Health Sciences, University of Catanzaro Magna Graecia, Catanzaro, Italy; ^2^ Unit of Psychiatry, Department of Medical Sciences and Public Health, University of Cagliari, Cagliari, Italy; ^3^ Department of Clinical Psychology, University Giustino Fortunato, Benevento, Italy; ^4^ Unit of Clinical Psychiatry, University Hospital Agency of Cagliari, Cagliari, Italy; ^5^ Department of Pharmacology, Dalhousie University, Halifax, NS, Canada

**Keywords:** bipolar disorder, affective temperament, treatment adherence, self-care, therapy

## Abstract

**Background:**

Affective temperament, defined as the fundamental predisposition from which normal affective states originate or as the constitutional core of personality, play a crucial role in mood disorders, particularly bipolar disorders. Understanding the relationship between temperaments, treatment adherence, and self-care is crucial for effective management and improved clinical results.

**Objectives:**

This study aims to (1) assess the correlation between affective temperaments and treatment adherence, (2) investigate the relationship between affective temperaments and self-care abilities, (3) identify predictors of treatment adherence, and (4) explore the moderating effect of self-care on the relationship between treatment adherence and depressive temperament in individuals with bipolar disorder.

**Methods:**

A cross-sectional study was conducted with 231 individuals diagnosed with bipolar disorder (BD) type I (N=160) and type II (N=71). The participants were evaluated using the following psychometric tools: Temperament Evaluation of Memphis, Pisa, and San Diego (TEMPS) to assess affective temperaments, Personal and Social Performance Scale (PSP) to evaluate social functioning and self-care abilities, and Morisky Medication Adherence Scale (MMAS) to measure treatment adherence. The study involved statistical analyses to examine correlations, identify predictors, and explore moderating effects.

**Results:**

The findings revealed significant correlations between affective temperaments and both treatment adherence and self-care abilities. Specifically, hyperthymic temperament was positively associated with higher treatment adherence, whereas cyclothymic and depressive temperaments were linked to lower adherence. Self-care abilities were found to mediate the relationship between depressive temperament and treatment adherence, suggesting that improved self-care can enhance adherence in individuals with depressive temperament.

**Conclusions:**

Affective temperaments significantly influence treatment adherence and self-care abilities in individuals with bipolar disorder. The mediating role of self-care highlights the importance of developing targeted interventions to improve self-care practices, thereby enhancing treatment adherence and overall well-being. Personalized treatment strategies based on temperament assessments could lead to better clinical outcomes and quality of life for individuals with bipolar disorder.

## 1 Introduction

Temperament encompasses the innate traits and characteristics shaped by genetic factors and biological structures, which remain largely unchanged throughout an individual’s lifetime. The impact of affective temperaments on the clinical manifestation, progression, and therapeutic outcomes is substantial, especially in the context of bipolar disorder and major depressive disorder. A predisposition towards a particular temperament in individuals with bipolar disorder markedly influences their insight, as well as their social, occupational, and familial functioning across various stages of the disorder (1). Recently, psychiatrists have shown increasing interest in studying the temperamental traits of individuals with mood conditions, particularly bipolar disorders (BD), and how these traits relate to affective symptoms (2). The convergence of individual temperaments, personality traits, and psychopathological features gives rise to a myriad of complex clinical phenotypes within the framework of mood disorders (3).

Temperament, often construed as the biological and enduring core of personality, serves as a crucial interface between the biological underpinnings and psychological manifestations of affective disorders (4). Defined as relatively stable traits, temperaments include various emotional dimensions, activity levels, social and biological rhythms, mood dispositions, and diurnal variations (5, 6). Kraepelin’s seminal work in 1921 laid the foundation for understanding a continuum between affective temperaments and affective pathologies, positing that fundamental affective states such as cyclothymic, irritable, anxious, and depressive temperaments precede the onset of full-blown manic-depressive episodes (6). Building upon Kraepelin’s insights, Akiskal refined the conceptualization of affective temperaments, delineating five primary types: a) Depressive temperament, characterized by introversion, depressed mood, lethargy, and hypersomnia; b) Cyclothymic temperament, typified by rapid mood oscillations and emotional lability; c) Hyperthymic temperament, marked by extroversion, heightened energy levels, and diminished sleep requirements; d) Irritable temperament, involving a propensity for quarrelsomeness and aggression (7) and e) Anxious-phobic temperament, characterized by persistent anxiety and excessive apprehension. This continuum perspective significantly influences current approaches to diagnosing and understanding mood disorders (8–10). Temperamental dysregulation serves as the pathological cornerstone of mood disorders, with deviations from normative temperament profiles indicating an elevated predisposition to developing mood disturbances, including BD (11). Extensive research has unveiled intriguing associations between affective temperaments and mood disorders. For instance, hyperthymic temperament is closely linked with BD-I and correlates with euphoria, psychomotor acceleration, reduced sleep duration, and increased frequency of manic episodes and hospitalizations. Conversely, cyclothymic temperament is associated with BD-II, anxiety disorders, and agoraphobia, while depressive temperament strongly correlates with depressive symptomatology. Additionally, irritable temperament has been specifically implicated in suicide risk (12, 13). These findings underscore the role of affective temperaments in shaping the clinical trajectory of mood disorders, particularly BD, acting as vulnerability factors, modifiers of clinical course, and influencers of patient insight (14, 15). Consequently, temperament assessment holds promise for improving treatment planning and optimizing clinical outcomes (16). BD are severe, recurrent psychiatric condition characterized by severe mood alterations, including depressive, manic, or hypomanic episodes, interspersed with periods of remission or relative stability (17, 18). These disorders significantly impact individuals’ physical and functional capacities, psychological health, and social interactions (19, 20). BD’s high prevalence and diverse manifestations contribute to its heterogeneity, posing challenges for accurate diagnosis and effective treatment (21, 22). A comprehensive evaluation of affective temperaments and their clinical correlates can aid in selecting tailored treatment modalities (23). Despite progress in elucidating the interplay between affective temperaments and symptomatology in BD, a full understanding of these connections remains elusive (24). Exploring affective temperaments in individuals with BD offers a rich and multifaceted area of inquiry, promising advancements in understanding the disorder’s etiology, course, and treatment. In addition, a frequent problem in patients with BD is non-adherence to treatment which is associated with high rates of recurrence and hospitalization, social functioning compromise, and reduced quality of life (25, 26). However, optimal medication management is not sufficient to prevent mood-related episodes, and psychosocial functioning and quality of life may continue to be compromised (27, 28). Self-care has become an important dimension in the healthcare continuum (29). In this regard, recommendations for supported self-management are included in international guidelines for the clinical treatment of BD (e.g (30), to enable individuals to better cope with symptoms and improve their quality of life (31).

The ongoing research into temperament and its clinical implications presents significant opportunities for developing targeted interventions to reduce the impact of BD and enhance the lives of those affected. This study examines how temperaments affect self-care and medication adherence in individuals with BD, considering their essential role in the disorder’s progression. Specifically, the present study aims to:

- Assess affective temperaments and their correlation with treatment adherence.
- Investigate the relationship between affective temperaments and self-care abilities.
- Identify predictors of treatment adherence.
- Explore the moderating effect of self-care on the relationship between treatment adherence and depressive temperament.

Understanding the synergistic effects of these temperamental influences is paramount for the effective management of BD. By enhancing clinical outcomes, improving the quality of life for patients, and reducing the societal burden of this chronic mental illness, targeted interventions based on temperament can lead to substantial benefits. The insights gained from this study could inform the development of more personalized treatment strategies, fostering better adherence to therapeutic regimens and promoting overall well-being among those affected by bipolar disorder.

## 2 Materials and methods

The study was observational, cross-sectional, and conducted in a naturalistic setting. Patients with BD were consecutively recruited from the Psychiatry Department of the “Magna Graecia” University of Catanzaro. Each participant received comprehensive information about the research protocol’s purpose, data protection measures, and the maintenance of privacy and anonymity. Participation was voluntary and required formal consent.

1. Inclusion criteria
  Age between 18 and 65 years, with the ability to read and understand the informed consent form.
2. Capability to answer self-report questionnaires.
3. Diagnosis of BD-I or BD-II.
4. Clinical stability at the time of enrollment, indicated by a Clinical Global Impression for Bipolar Patients (CGI-BP) score of ≤2 on item 1 (severity of illness).
5. Stability under therapy for at least six months.

Exclusion criteria

1. Patient refusal to participate.
2. Presence of major neurological conditions, such as epilepsy, cognitive impairment, or genetic syndromes with psychiatric manifestations.
3. Psychiatric comorbidity or any condition preventing the completion of the global evaluation, such as language barriers and severe cognitive disabilities.

Upon agreeing to participate and signing a written consent form, participants underwent a series of clinical and psychopathological assessments. These evaluations were conducted at the conclusion of outpatient clinical visits, with scale administration occurring either at the end of these visits or at separate times. Psychometric scale assessments and demographic data collection were carried out by researchers, specialist psychology trainees, and research fellows.

The diagnosis of BD was established according to the criteria outlined in the Diagnostic and Statistical Manual of Mental Disorders, Fifth Edition (DSM-5) (32), utilizing the Structured Clinical Interview for DSM-5 Disorders, Clinician Version (SCID-5-CV) (33). Each enrolled patient participated in a semi-structured clinical interview to obtain comprehensive clinical and anamnestic information. Sociodemographic and clinical data were gathered using a department-developed questionnaire, divided into two sections. The first section focused on sociodemographic data, including life history, age, gender, marital status, education level, type of occupation, family history of other illnesses, and comorbidities. The second section collected detailed psychiatric history, encompassing diagnosis, family history, onset and course characteristics, illness duration, age at first episode onset, lifetime prevalence, hospitalizations (including total number and average duration), current pharmacological treatment, and information on previous suicide attempts. In line with the Ethical Committee’s guidelines, participants received a comprehensive explanation of the study’s aims and methods and provided written informed consent be-fore any procedures. The study protocol was submitted to and approved by the Ethical Committee of the University Hospital Mater Domini in Catanzaro (n.307/2020). The study was conducted in accordance with the ethical principles of the revised Helsinki Declaration.

### 2.1 Psychometric tools

The Temperament Evaluation of Memphis, Pisa, and San Diego (TEMPS) scale was employed to assess affective temperaments. Initially developed as an interview (TEMPS-I) and later as a self-administered questionnaire (TEMPS-A), TEMPS-A consists of 110 items with dichotomous “yes” or “no” responses (34, 35). Erfurth et al. (36) developed a shorter version, TEMPS-M, based on TEMPS-A, which includes 35 items scored on a Likert scale from 1 to 5, facilitating a dimensional exploration of the assessed domains.

The Clinical Global Impression for Bipolar Patients (CGI-BP) is a modified version of the original CGI, specifically designed to evaluate global illness severity and changes in patients with bipolar disorder. It has been utilized with Italian clinical samples and is divided into two sections: severity of illness and global improvement. Both sections are scored from 1 (“normal, not ill at all”) to 7 (“among the most extremely ill patients”), with 0 indicating the impossibility of assessment (37).

The Morisky Medication Adherence Scale (MMAS) was used to assess patients’ medication-taking behavior. This scale evaluates various factors related to medication adherence, such as forgetting to take medications, not bringing medications when traveling, not taking medications when feeling worse or better, and difficulty in continuing drug treatment plans. Scores range from 0 to 8, with higher scores indicating better treatment adherence (38–40).

The Personal and Social Performance Scale (PSP), originally developed by Morosini et al. (41), measures the routine social functioning of patients with psychiatric disorders. The PSP assesses four main areas: (a) socially useful activities, (b) personal and social relationships, (c) self-care, and (d) disturbing and aggressive behaviors. Two different sets of operational criteria are used to judge the degree of difficulties: one for areas a-c and another specific to area d, disturbing and aggressive behavior. The degrees of severity are classified as: (I) absent, (II) mild, (III) manifest but not marked, (IV) marked, (V) severe, and (VI) very severe. The PSP is a 100-point single-item rating scale subdivided into 10 equal intervals (41). Validated in terms of its psychometric properties, the PSP allows for easy and rapid administration, making it frequently used in pharmacological settings to assess the impact of therapy, particularly antipsychotics, on individual functioning.

For this study, we specifically utilized the sub-item “self-care” due to its challenging nature in evaluating bipolar patients’ ability to manage daily activities, which is often linked to poor medical compliance. According to our hypothesis, it is precisely the lack of self-care that reflects hopelessness and significantly undermines treatment adherence. Additionally, we opted not to use all items or the total score, as some were already covered in the self-assessment conducted, including aspects such as aggressive behavior, interpersonal relationships, and social functioning.

### 2.2 Statistical analysis

Statistical analyses were conducted using the Statistical Package for Social Sciences Version 26 (SPSS, Chicago, Illinois, USA). Descriptive analyses were performed to assess the distribution of variables across the entire sample, with results reported as frequencies for categorical variables and as means ± standard deviations (SD) or medians for continuous variables. To test the specified hypotheses, the following analyses were conducted:

Assessment of affective temperaments and their correlation with treatment adherence and self-care abilities:

- Correlation analyses were performed to evaluate the relationships between different types of affective temperaments and both treatment adherence and self-care abilities.
- Two regression analyses were conducted:

To examine the association between affective temperaments and self-care abilities, a linear regression analysis was used where self-care ability, obtained from the PSP rating scale, was entered as a continuous dependent variable.

To estimate factors associated with treatment adherence, a binomial logistic regression analysis was performed. The ‘treatment adherence’ variable was set as a dichotomous dependent variable, and affective temperaments were included as independent variables.

Moderation analysis:

- To explore the moderating effect of self-care on the relationship between treatment adherence and temperament, a moderation analysis was performed. In this analysis, the depressive temperament served as the dependent variable, the self-care item of the PSP assessment scale as the moderator, and the treatment adherence item of the MMAS scale as the predictor.

## 3 Results

### 3.1 Sociodemographic and clinical characteristics of the sample

The final sample comprised 231 patients diagnosed with BD, including 112 males (48.5%) and 119 females (51.5%), with a mean age of 46.85 years (± 13.87). The most prevalent diagnosis was BD-I (69.3%, 160), with an average age of onset at 26.04 years (± 9.46). Most of the participants were female and had completed high school (73.2%, 169). Regarding clinical characteristics, a significant proportion of the sample had a family history of psychiatric illness (58.8%, 134), mixed features (55.0%, 127), anxious features (58.9%, 136), aggression (56.7%, 131), psychotic symptoms (42.8%, 98), and exhibited a seasonal pattern (50.9%, 115). A smaller percentage had attempted suicide (29.4%, 68). Participants reported an average of 5.70 (± 5.70) depressive episodes, 4.00 (± 4.16) manic episodes, and 2.79 (± 2.76) hypomanic episodes, with the average number of affective episodes being 11.17 (± 9.75). Additionally, around 35.1% (81) reported a history of drug abuse (45 cannabis; 26 cocaine; 10 alcohol).

**Table 1** presents the mean scores obtained from various instruments used in the study. The mean scores of affective temperaments, as measured by the Brief TEMPS-M, were as follows: depressive temperament 22.27 (± 6.68), hyperthymic temperament 20.23 (± 6.31), anxious temperament 18.75 (± 5.91), cyclothymic temperament 22.14 (± 7.49), and irritable temperament 20.10 (± 7.71). The mean values of the different areas examined by the PSP were: socially useful activities 2.29 (± 0.65), interpersonal relationships 2.78 (± 0.87), self-care 3.60 (± 1.48), and aggressive behaviors 3.03 (± 1.24). The administration of the MMAS revealed an average score of 2.51 (± 1.054).

**Table 1:** Socio-demographic and clinical variables.

Characteristics	Sample = 231
Sex M, N (yes%)	112 (48,5%)
Age, M, (SD±)	46,85 (± 13,871)
Age of onset, M, (SD±)	26,04 (± 9,463)
Married, N (yes%)	122 (52,8%)
High school degree, N (yes%)	169 (73.2%)
Bipolar disorder type I N (yes%)	160 (69,3%)
Bipolar disorder type II N (yes%)	71 (30,7%)
Familial history of psychiatric disorders, N (yes%)	134 (58,0%)
Suicide attempt, N (yes%)	68 (29,4%)
Mixed features, N (yes%)	127 (55,0%)
Anxious features, N (yes%)	136 (58,9%)
Number of total episodes, M, (SD±)	11,17 (± 9,749)
Number of depressive episodes, M, (SD±)	5,70 (± 5,699)
Number of manic episodes, M, (SD±)	4,00 (± 4,157)
Number of hypomanic episodes, M, (SD±)	2,79 (± 2,764)
Duration of the disease in years, M, (SD±)	20,81 (± 12,759)
Seasonality, N (yes%)	115 (50,9%)
Psychotic symptoms, N (yes%)	98 (42,8%)
Aggressiveness, N (yes%)	131 (56,7%)
Lifetime abuse, N (yes%)	81 (35,1%)
MMAS adherence M, (SD±)	2,51 (± 1,054)
Depressive, M, (SD±)	22,27 (± 6,676)
Hyperthymic, M, (SD±)	20,23 (± 6,306)
Anxious, M, (SD±)	18,75 (± 5,912)
Cyclothymic, M, (SD±)	22,14 (± 7,494)
Irritable, M, (SD±)	20,10 (± 7,708)
Useful activities, M, (SD±)	2,29 (± 0,646)
Interpersonal relations, M, (SD±)	2,78 (± 0,873)
Self-care, M, (SD±)	3,60 (± 1,476)
Aggressive behaviors, M, (SD±)	3,03 (± 1,243)

MMAS: Morisky Medication Adherence Scale; Self-care, Useful activities, Interpersonal relations, Aggressive behaviors: PSP items.

### 3.2 Assessment of affective temperaments and their correlation with treatment adherence

Spearman correlation analysis (**Table 2**) revealed statistically significant correlations (p<0,01) between various affective temperaments, MMAS scores, and the domains assessed by the PSP. The results indicated that all affective temperaments, except for the hyperthymic temperament, had significant negative correlations with the total MMAS score. In contrast, the hyperthymic temperament demonstrated a positive correlation with the MMAS score (p<0,01). Positive correlations were observed between all temperaments and self-care, except for the hyperthymic temperament, which showed a negative correlation in this domain (p<0,01). Further analysis of the PSP areas revealed that the hyperthymic temperament had negative correlations with socially useful activities (p<0,01) and aggressive behaviors (p<0,01). The cyclothymic temperament positively correlated with interpersonal relationships (p<0,01), while the irritable temperament showed a positive correlation with aggressive behaviors (p<0,01). Notably, the depressive temperament had significant correlations with all factors considered in the analysis, including the various PSP domains. This comprehensive association underscores the pervasive impact of depressive temperament across multiple aspects of functioning and adherence.

**Table 2:** Correlation between affective temperaments, MMAS and PSP items.

	Depressive	Hyperthymic	Anxious	Cyclothymic	Irritable	MMAS adherence	Useful activities	Interpersonal relations	Self-care	Aggressive behaviors
**Depressive**	1	-,291**	,261**	,492**	,369**	-,647**	,207**	,207**	,738**	,325**
**Hyperthymic**	-,291**	1	0,008	0,007	-0,028	,192**	-,389**	0,004	-,265**	-,135*
**Anxious**	,261**	0,008	1	,308**	,133*	-,256**	-0,001	0,104	,157*	0,026
**Cyclothymic**	,492**	0,007	,308**	1	,150*	-,221**	0,107	,495**	,280**	0,109
**Irritable**	,369**	-0,028	,133*	,150*	1	-,301**	,137*	0,012	,310**	,645**
**MMAS** **adherence**	-,647**	,192**	-,256**	-,221**	-,301**	1	-0,107	-0,012	-,676**	-,194**
**Useful** **activities**	,207**	-,389**	-0,001	0,107	,137*	-0,107	1	0,075	,242**	0,031
**Interpersonal relations**	,207**	0,004	0,104	,495**	0,012	-0,012	0,075	1	,186**	0,075
**Self-care**	,738**	-,265**	,157*	,280**	,310**	-,676**	,242**	,186**	1	,283**
**Aggressive** **behaviors**	,325**	-,135*	0,026	0,109	,645**	-,194**	0,031	0,075	,283**	1

MMAS, Morisky Medication Adherence Scale; Self-care, Useful activities, Interpersonal relations, Aggressive behaviors: PSP items.

**The correlation is significant at the 0.01 level (Two-tailed). *The correlation is significant at the 0.05 level (Two-tailed).

### 3.3 Investigation of the relationship between affective temperaments and self-care abilities

A linear regression analysis was performed to explore the relationship between affective temperaments and self-care abilities, adjusting for sex and age. The self-care item of the PSP served as the continuous dependent variable, while the five affective temperaments were the independent variables (**Table 3**). The results revealed a significant association between poor self-care and depressive temperament (p = 0.001), underscoring the profound impact of depressive temperament on an individual’s ability to manage daily self-care tasks.

**Table 3:** Regression analysis with dependent variable self-care.

Coefficients					
Model	Non-standardized coefficients		Standardised coefficients	t	Sign.
	B	Standard Error	Beta		
**(Constant)**	,652	,444		1,468	,143
**Depressive**	,168	,013	,759	13,016	,001
**Hyperthymic**	-,008	,011	-,034	-,711	,478
**Anxious**	-,004	,012	-,015	-,306	,760
**Cyclothymic**	-,017	,011	-,085	-1,597	,112
**Irritable**	,008	,009	,042	,883	,378

### 3.4 Identification of factors associated with treatment adherence

A binomial logistic regression was conducted to identify factors associated with treatment adherence using the Morisky Medication Adherence Scale (MMAS) rating “treatment adherence” as the dependent variable (**Table 4**). Treatment adherence was set as a dichotomous variable (absence/presence of treatment adherence). Previous correlation analyses had indicated significant associations between treatment adherence and various temperaments. The regression analysis confirmed a statistically significant association between poor adherence and both depressive and cyclothymic temperaments (p < 0.001). Notably, depressive temperament emerged as a factor strongly associated with poor treatment adherence, highlighting its critical role in influencing patient compliance with treatment regimens.

**Table 4:** Logistic regression analysis.

Variables in the equation									
	B	S.E.	Wald	gl	Sgn.	Exp(B)	95% C.I. per EXP(B)	
							Inferior	Superior
**Depressive** ^a^	-,385	,049	62,182	1	,001	,681	,619	,749
**Cyclothymic** ^b^	,103	,030	11,402	1	,001	1,108	1,044	1,176
**Constant**	6,628	,969	46,792	1	,000	755,676		

a. Variables included in phase 1: depressive; b. Variables included in phase 2: cyclothymic.

### 3.5 Analysis of the moderating effect of self-care on the relationship between treatment adherence and depressive temperament

**Figure 1:**
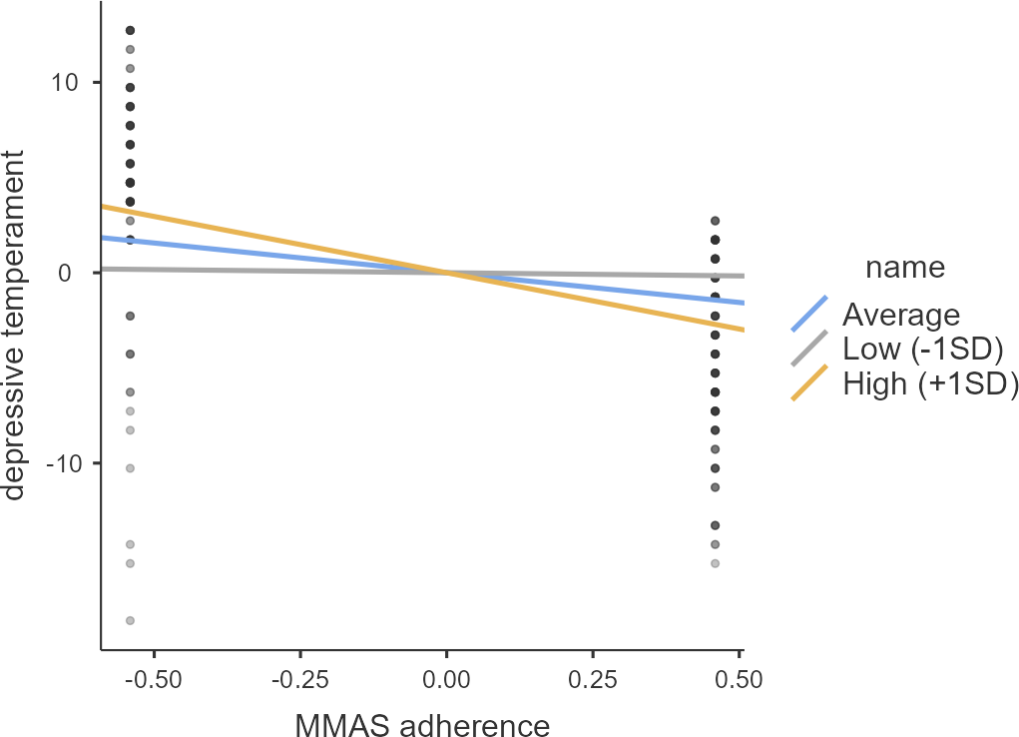
Moderation analysis (Simple Slope Plot).

A moderation analysis was conducted to investigate the moderating effect of self-care on the relationship between treatment adherence and depressive temperament. In this analysis, treatment adherence was set as the independent variable, depressive temperament as the dependent variable, and self-care as the moderator (**Table 5**). The results, depicted in **Table 6** and **Figure 1**, demonstrate that the effects of treatment adherence on depressive temperament were significant (p < 0.001) at both high (+1 SD) and average levels of the moderator variable. These findings indicate that the impact of treatment adherence on depressive temperament is significantly moderated by self-care, particularly at high and average levels (p < 0.001).

**Table 5:** Moderation analysis between depressive temperament, MMAS, and Self-care.

Moderation Estimates				
	Estimate	SE	Z	P
**MMAS** **adherence**	-3.12	0.571	-5.47	< .001
**Self-care**	2.39	0.191	12.52	< .001
**MMAS** **adherence ✻ Self-care**	-1.89	0.535	-3.54	< .001

MMAS: Morisky Medication Adherence Scale; Self-care: PSP item.

**Table 6:** Simple slope estimates.

Simple Slope Estimates				
	Estimate	SE	Z	p
**Average**	-3.122	0.600	-5.205	< .001
**Low (-1SD)**	-0.333	1.070	-0.311	0.756
**High (+1SD)**	-5.911	0.923	-6.403	< .001

Shows the effect of the predictor (MMAS adherence) on the dependent variable (Depressive temperament) at different levels of the moderator (Self-care).

A similar moderation analysis was performed using cyclothymic temperament as the independent variable. However, this analysis did not yield statistically significant results.

## 4 Discussion

The present study elucidates the intricate interplay between affective temperaments, self-care, and medication adherence among individuals diagnosed with BD. Managing BD presents numerous challenges, particularly in maintaining consistency in drug therapy. This study explores largely uncharted territory by examining how different temperamental profiles influence self-care behavior and adherence to drug regimens. This investigation is crucial as treatment discontinuation often leads to unfavorable outcomes, highlighting the urgent need to identify potential predictors of poor adherence (42). Affective temperaments encompass a spectrum of mood states, including depressive, hyperthymic, cyclothymic, anxious, and irritable temperaments, each exerting unique influences on an individual’s responses to environmental stimuli (9). Understanding their role in shaping treatment-related behaviors is paramount for tailoring interventions to individuals; inherent traits and optimizing medication outcomes (43). Our findings highlight the significant impact of depressive temperament on both self-care abilities and treatment adherence. The pervasive impact of depressive temperament on multiple facets of daily functioning and health behaviors underscores the need for a nuanced approach to therapeutic interventions.

Furthermore, the study demonstrates the moderating effect of self-care on the relationship between treatment adherence and depressive temperament. This suggests that interventions aimed at improving self-care skills could mitigate the negative impact of depressive temperament on compliance, essential for improving the prognosis and quality of life for individuals with BD. Several previous studies have investigated the association between affective temperaments and pharmacological treatment adherence in individuals with BD, shedding light on the complex interplay between temperament and treatment response (12, 15, 42, 44–50). For example, research by Miola and colleagues found that individuals with a predominantly depressive temperament experienced more severe episodes and poorer treatment responses, highlighting the potential influence of temperament on treatment outcomes (51). Similarly, numerous studies have identified cyclothymic temperament as a predictor of poor outcomes in BD, characterized by increased symptom severity, higher rates of relapse, hospitalization, and suicide attempts (12, 44, 48, 52–55). Our findings build upon this existing literature by revealing depressive temperament as a significant mediating factor in compromised self-care and treatment adherence among people with BD. The prolonged duration of depressive episodes and the increased susceptibility to hopelessness associated with this temperament are likely to lead to treatment discontinuation and self-medication, particularly with substances such as alcohol. This emphasizes the clinical importance of considering affective temperaments in BD before treatment planning and intervention development. Moreover, our study identifies significant negative correlations between all affective temperaments, except hyperthymic, and medication adherence, as measured by the Morisky MMAS. These findings suggest that individuals with depressive, anxious, cyclothymic, and irritable temperaments may be more likely to exhibit poor medication compliance, highlighting the need for tailored interventions to address temperament-specific challenges in treatment adherence. This result might seem contradictory, as some studies have demonstrated that hyperthymic temperament often predisposes individuals to greater symptom severity. However, this discrepancy can be explained by the fact that, although patients with a hyperthymic temperament may experience more intense episodes, those with depressive, anxious, or cyclothymic temperaments have a more nuanced symptomatology that responds poorly to stabilizers and remain more stable over time.

Further exploration reveals the comprehensive association between depressive temperament and various domains assessed by the PSP scale, including socially useful activities, interpersonal relationships, self-care, and aggressive behaviors. Consistent with prior research, the present findings underscore the pervasive impact of depressive temperament on different facets of social and personal functioning in people with BD (42, 55). Conversely, hyperthymic temperament exhibits positive correlations with medication adherence and negative correlations with aggressive behaviors while being associated with helpful activities among individuals with this temperament, indicative of a more extroverted and energetic disposition (9). Additionally, the cyclothymic temperament is associated with both medication compliance and self-care, albeit to a lesser extent than the depressive temperament, suggesting variability in adherence behaviors among individuals with cyclothymic tendencies (53).

Logistic regression analyses identify depressive and cyclothymic temperaments as significant predictors of poor treatment adherence, highlighting their clinical relevance in predicting medication compliance in BD. Moderation analysis further emphasizes the importance of depressive temperament, suggesting that targeted self-care interventions could enhance medication compliance in these individuals. The current study contributes to the existing literature by elucidating the nuanced connections between affective temperaments, treatment adherence, and self-care abilities in individuals with BD. The moderation estimates presented reveal significant insights into the interplay between treatment adherence, self-care, and their combined effect on depressive temperament. The adherence estimate derived from the MMAS (-3.12) indicates a significant negative impact, particularly in individuals with a depressive temperament. This underscores the urgent need for rigorous pharmacological monitoring, timely intervention, and the application of tailored psychoeducational strategies to ensure proper medication adherence. On the other hand, the estimate for Self-Care (2.39) demonstrates a significant positive effect, indicating that individuals who exhibit better self-care abilities tend to experience lower levels of depressive temperament. This highlights the crucial role of self-care practices in promoting mental well-being and reducing depressive tendencies. The most intriguing result emerges from the interaction term MMAS Adherence and Self-Care (-1.89). This estimate suggests a moderating effect wherein the combined influence of treatment adherence and self-care on depressive temperament is notable. Specifically, it implies that while both treatment adherence and self-care independently contribute to lower levels of depressive temperament, their joint effect is even more pronounced. This finding demonstrates the synergistic relationship between treatment adherence and self-care in alleviating depressive symptoms. Overall, these results underscore the multifaceted nature of depressive temperament and the importance of considering both treatment adherence and self-care practices in managing and preventing depressive symptoms. The findings highlight the need for comprehensive interventions that address not only medication adherence but also emphasize the importance of fostering self-care behaviors to effectively mitigate depressive tendencies and enhance overall mental well-being. In particular, psychosocial therapies effectively improve medication adherence, identify early warning signs, and increase self-management skills (56). Among the psychosocial interventions, we have cognitive behavioral therapy (CBT) (57, 58), psychoeducation (PE) (59–62), family-focused therapy (FFT), and interpersonal and social rhythm therapy (IPSRT) (63–66). In addition, one way to reduce the risk of poor adherence to interventions could be to assess affective temperament early on since it is a vulnerability factor, influences the clinical picture, and can change the course of the disease. This could be useful to improve the planning of the treatment plan.

While the current study provides valuable insights, several limitations warrant consideration. The cross-sectional design limits our ability to establish causality or delineate temporal relationships between affective temperaments, treatment adherence, and self-care. Future longitudinal studies are needed to elucidate the dynamic interaction of these factors over time. Additionally, reliance on self-reported measures of medication adherence and self-care introduces potential recall bias and social desirability effects. Incorporating objective measures, such as electronic medication monitoring, may enhance the accuracy of adherence assessments. Further studies should incorporate a specific assessment for personality disorders and confirm syndromic stability using designated rating scales for depressive and manic symptoms, such as the Montgomery-Asberg Depression Rating Scale or the Young Mania Rating Scale. Additionally, more precise assessment instruments could be employed alongside the PSP. The generalizability of our findings may be influenced by the specific demographic and clinical characteristics of the studied sample, necessitating the inclusion of a more diverse and representative population to bolster external validity. Moreover, future research should consider various additional factors that may affect treatment adherence, including hospitalization, the involvement of a caregiver, and participation in a psychoeducation group. Despite these limitations, the findings not only enhance our understanding of the nuances involved but also lay the groundwork for future research, paving the way for more tailored and effective interventions.

## Data Availability

The raw data supporting the conclusions of this article will be made available by the authors, without undue reservation.
